# DENV-1 Infection of Macrophages Induces Pyroptosis and Causes Changes in MicroRNA Expression Profiles

**DOI:** 10.3390/biomedicines12122752

**Published:** 2024-11-30

**Authors:** Qinyi Zhang, Sicong Yu, Zhangnv Yang, Xingxing Wang, Jianhua Li, Lingxuan Su, Huijun Zhang, Xiuyu Lou, Haiyan Mao, Yi Sun, Lei Fang, Hao Yan, Yanjun Zhang

**Affiliations:** 1 Zhejiang Provincial Center for Disease Control and Prevention, Hangzhou 310051, China; 2 School of Medical Technology and Information Engineering, Zhejiang Chinese Medical University, Hangzhou 310053, China; 3 The First People’s Hospital of Xiaoshan District, Hangzhou 311201, China; 4 Key Laboratory of Public Health Detection and Etiological Research of Zhejiang Province, Hangzhou 310051, China; 5 Institute of Laboratory Animal Sciences, Chinese Academy of Medical Sciences & Peking Union Medical College, National Human Diseases Animal Model Resource Center, Beijing 100021, China; 6 Department of Critical Care Medicine, Sir Run Run Shaw Hospital, College of Medicine, Zhejiang University, Hangzhou 310016, China; 7 Key Laboratory of Microbial Technology and Bioinformatics of Zhejiang Province, Hangzhou 310016, China

**Keywords:** dengue virus, macrophages, pyroptosis, microRNA

## Abstract

Background: Dengue virus (DENV) is the most widespread mosquito-borne virus, which can cause dengue fever with mild symptoms, or progress to fatal dengue hemorrhagic fever and dengue shock syndrome. As the main target cells of DENV, macrophages are responsible for the innate immune response against the virus. Methods: In this study, we investigated the role of pyroptosis in the pathogenic mechanism of dengue fever by examining the level of pyroptosis in DENV-1-infected macrophages and further screened differentially expressed microRNAs by high-throughput sequencing to predict microRNAs that could affect the pyroptosis of the macrophage. Results: Macrophages infected with DENV-1 were induced with decreased cell viability, decreased release of lactate dehydrogenase and IL-1β, activation of NLRP3 inflammasome and caspase-1, cleavage of GSDMD to produce an N-terminal fragment bound to cell membrane, and finally induced macrophage pyroptosis. MicroRNA expression profiles were obtained by sequencing macrophages from all periods of DENV-1 infection and comparing with the negative control. Sixty-three microRNAs differentially expressed in both the early and later stages of infection were also identified. In particular, miR-223-3p, miR-148a-3p, miR-125a-5p, miR-146a-5p and miR-34a-5p were recognized as small molecules that may be involved in the regulation of inflammation. Conclusions: In summary, this study aimed to understand the pathogenic mechanism of DENV through relevant molecular mechanisms and provide new targets for dengue-specific therapy.

## 1. Introduction

Dengue virus (DENV) is currently considered to be the most dominant mosquito-borne virus affecting human health, causing more than 100 million confirmed infections in 125 countries each year, with about 10,000 deaths reported annually. The mortality rate related to DENV has been increasing in recent years [1,2]. DENV is mainly transmitted by the bites of *Aedes aegypti* and *Aedes albopictus* mosquitoes, and it is generally not transmitted directly from person to person. Clinical manifestations range from asymptomatic infected cases to severe dengue fever, which can be fatal. Dengue fever (DF) is a mild influenza-like syndrome, including fever, nausea, vomiting, rash, pain, lymph node swelling and leukopenia. In severe cases, it can lead to dengue hemorrhagic fever (DHF) or even dengue shock syndrome (DSS), with a mortality rate of up to 20% [3]. As a mosquito-borne virus, the unique transmission route of DENV makes it mainly occur in tropical and subtropical regions where mosquitoes are widely distributed. However, due to global warming, the spread of DENV has intensified, leading the incidence rate of DF to increase year by year. Therefore, more and more populations are exposed to the risk of DENV infection and preventing and controlling the transmission of DENV infection has become an international public safety issue [4,5].

DENV belongs to the *flavivirus* family and is characterized as an enveloped, single-stranded, positive-sense RNA virus with viral particles ranging from 45 to 55 nm in diameter and a genome length of 10.7 kb. DENV infection causes the body to produce excessive pro-inflammatory cytokines, resulting in increased endothelial cell permeability and impaired endothelial integrity, as well as aggravating symptoms in DF cases [3]. Therefore, ameliorating exacerbated inflammation is an important goal of treatment. Macrophages, as the primary target cells for DENV, are also the key immune cells responsible for releasing pro-inflammatory cytokines, such as interleukin-1β (IL-1β), which is considered to be an essential factor in regulating vascular integrity [6]. Pyroptosis, as a mode of programmed cellular inflammatory death, differs from apoptosis in that it does not preserve the integrity of the cell membrane. It is characterized by the activation of the inflammasome, persistent membrane swelling and rupture, and it then releases IL-1β, which exacerbates the inflammatory response [7]. Previous studies have shown that DENV-2 activates the NOD-like receptor protein 3 (NLRP3) inflammasome via C-type lectin 5A (CLEC5A) in human macrophages, inducing high levels of IL-1β and IL-18 expression, while caspase-4 is involved in the regulation of pyroptosis upstream of caspase-1 [8,9].

MicroRNA (miRNA) is a class of non-coding single-stranded RNA molecules encoded by endogenous genes with a length of about 22 nucleotides, which plays an important role in the regulation of gene expression and is intricately linked to a multitude of life processes, such as growth and development, cellular differentiation, apoptosis, antiviral response and antitumor response [10]. Meanwhile, miRNA can participate in regulating inflammasome activation and pyroptosis. For example, miR-195 was significantly downregulated in SH-SY5Y human neuroblastoma cells infected with enterovirus A71, ultimately demonstrating that miR-195 regulates EV-A71-induced pyroptosis by directly targeting NLRX1 [11]. This provided a new direction for studying DENV-induced macrophage pyroptosis.

Analyzing the miRNA transcriptome to screen for differentially expressed miRNAs and enrichment pathways is a powerful means of revealing the molecular mechanisms of miRNAs in biological processes and disease development. There are two main methods for miRNA transcriptome analysis: high-throughput sequencing [12] and microarray technology [13]. Microarray technology uses pre-designed labeled probes to hybridize with cDNA sequences, while sequencing does not rely on pre-designed probes or known sequences and can cover a wider range of genomes.

Currently, it has been found that miRNAs can regulate DENV replication by targeting the DENV genome or host genes [14,15]. Vascular dysfunction is one of the main causes of severe dengue fever. PPARγ regulates the downregulation of miR-573 after DENV infects vascular endothelial cells and protects endothelial function [16]. In addition, miR-383-5p inhibits DENV replication in liver cells [17]. However, the research on DENV-induced pyroptosis is still limited and the regulatory role of miRNA in it requires further investigation.

In recent years, DENV-1 has been dominant in the local outbreaks of DF in Zhejiang Province, China [18], but the regulatory mechanism of DENV-1-induced macrophage pyroptosis is still unclear. Pyroptosis, as a mode of inflammatory cell death, can lead to excessive production of pro-inflammatory cytokines, possibly leading to vascular leakage and severe DHF/DSS. The aim of this study was to verify that DENV-1 infection induced pyroptosis in macrophages, exacerbating the inflammatory response. Furthermore, miRNA transcriptome sequencing technology was used to screen for differentially expressed miRNAs before and after infection to identify potential targets that regulate macrophage pyroptosis and inflammatory response.

## 2. Materials and Methods

### 2.1. Cell Culture

Human monocyte cell line THP-1 cells (MeisenCTCC, Jinhua, China) were cultured in Roswell Park Memorial Institute 1640 medium (RPMI 1640; Gibco, Grand Island, NY, USA), supplemented with 10% fetal bovine serum (FBS; Gibco, New York, NY, USA), 1% L-glutamine (LG; Gibco, USA) and 1% penicillin-streptomycin (PS; Gibco, USA), in a 37 °C, 5% CO_2_ incubator. THP-1 cells were stimulated using 10 ng/mL of phorbol-12-myristate-13-acetate (PMA; Sigma, Burlington, MA, USA) for 72 h to differentiate THP-1 cells into M0-type macrophages. The PMA-containing culture medium was removed and the cells were washed three times with phosphate-buffered saline (PBS; Gibco, USA), and RPMI 1640 (2% FBS, 1% LG and 1% PS) was added finally for subsequent experiments. C6/36 mosquito cells were preserved by the Zhejiang Provincial Center for Disease Control and Prevention, cultured in RPMI 1640 containing 10% FBS, 1% LG, 1% PS and 1% sodium pyruvate (SP; Gibco, USA) medium at 28 °C with 5% CO_2_.

### 2.2. Virus Amplification and Titration

The DENV-1 strain was isolated from a patient with dengue fever in Zhejiang Province, replicated and amplified in C6/36. When C6/36 was almost confluent into a monolayer of cells, 100 μL of serum sample was added and cultured for 7 days, then the cells were repeatedly freeze−thawed three times and centrifuged at 2000 rpm for 10 min to collect the supernatant as the first-generation viral culture, which was stored in portions at –80 °C. The above steps were repeated to obtain the second-generation viral culture, and it was stored at –80 °C separately. Viral RNA was extracted using the RNeasy Mini Kit (QIAGEN, Hilden, Germany) for nucleic acid extraction and detection. The One Step Primer^TM^ RT-PCR Kit (TaKaRa, Kusatsu, Japan) was combined with DENV-1-specific primer probes to detect viral RNA. The real-time RT-PCR reaction system and program were configured according to the manufacturer’s instructions.

In the meantime, the C6/36 cells were used to determine the titer of viral TCID_50_. The C6/36 cells were inoculated into 96-well plates (Corning Inc., Corning, NY, USA) and placed in an incubator for 24 h. When the cells grew to a monolayer, a viral strain dilution of RPMI 1640 diluted to 10^−1^, 10^−2^, 10^−3^, 10^−4^, 10^−5^, 10^−6^, 10^−7^, 10^−8^ was added to the 96-well plate at 100 μL per well and 100 μL of RPMI 1640 (2% FBS) was supplemented. After 5–7 days of incubation at 28 °C, in a 5% CO_2_ incubator, the number of wells with lesions was recorded and the TCID_50_ was calculated using the Reed–Muench method [19].

### 2.3. Cell Activity

THP-1 cell suspensions were inoculated into 96-well plates at 5 × 10^4^ per well and 100 μL per well, and differentiation was induced by PMA. DENV-1 was added to macrophages at multiplicities of infection (MOIs) of 0.01, 0.1 and 1. After adsorption for 2 h, the virus-containing culture medium was discarded, and the cells were washed with PBS twice. A total of 100 μL of RPMI 1640 (2% FBS) was added to each well, and the cells were incubated at 37 °C in a 5% CO_2_ incubator. Cells without any treatment were used as the negative control, and 1 mg/mL lipopolysaccharides (LPS; Sigma, USA) and 2 μM Nigericin (Sigma, USA) were treated for 4 h as the positive control. Using a CCK-8 cytotoxicity detection kit (MeilunBio, Dalian, China) at 24 h, 48 h and 72 h after viral infection, the old medium was discarded, and RPMI 1640 (2% FBS) containing 10% CCK-8 was added to each well. After incubation at 37 °C for 2 h, the absorbance value was measured by an enzyme marker at a primary wavelength of OD 450 nm, and a secondary wavelength of OD 600 nm, as the cell viability = (OD experimental group − OD blank)/(OD negative control − OD blank) × 100%.

### 2.4. Lactate Dehydrogenase (LDH) Release Assay

THP-1 cells were inoculated into 96-well plates, induced to differentiate with PMA and categorized into the infection group (MOIs = 0.01, 0.1, 1), negative control and positive control (LPS and Nigericin treatment) as described previously. An LDH assay kit (Beyotime, Shanghai, China) was used according to the manufacturer’s protocol, and 10% of the original volume of LDH-releasing reagent was added to the maximal enzymatic activity control wells one hour before 24 h, 48 h and 72 h of viral infection, respectively. The incubation was continued for 1 h. The 96-well plate was centrifuged at 400× *g* for 5 min, and 120 μL of the supernatant was transferred to a new 96-well plate and configured according to the instructions of the LDH working solution. Adding 60 μL of LDH working solution to each well, incubating for 30 min at room temperature and avoiding light then followed, along with measuring the absorbance values with an enzyme marker at a primary wavelength of OD 490 nm and a secondary wavelength of OD 600 nm. The OD value of each group was calculated after subtracting the blank control, according to the following: LDH release rate = (OD experimental group − OD negative control)/(OD maximal enzyme activity − OD negative control) × 100%.

### 2.5. Enzyme-Linked Immunosorbent Assay (ELISA) to Detect IL-1β

THP-1 cell suspensions were inoculated into six-well plates at 1 × 10^6^ cells per well and 2 mL per well and induced to differentiate with PMA. DENV-1 was infected as described previously and divided into the infected groups (MOIs = 0.01, 0.1, 1), negative control and positive control (LPS and Nigericin treatment). Cell supernatants were collected at the indicated times. The assay was performed using the human IL-1β ELISA kit (Abcam, Cambridge, UK), where 100 μL TMB developer was added according to the manufacturer’s protocol and incubated at room temperature in darkness for 10 min. After incubation, 100 μL of termination solution was added to each well to terminate the reaction. Finally, absorbance values were measured at OD 450 nm, and a standard curve was plotted to calculate the amount of IL-1β in each sample.

### 2.6. Real-Time Quantitative PCR (RT-qPCR)

THP-1 cells were inoculated into six-well plates, induced to differentiate with PMA, and divided into the infected groups (MOIs = 0.01, 0.1, 1), negative control and positive control (LPS and Nigericin treatment) as described previously. Cellular RNA was extracted at the indicated times using the nucleic acid extraction kit RNeasy Mini Kit (QIAGEN, Germany), and an 8 μL RNA template was reverse transcribed to synthesize cDNA (TaKaRa, Japan). After completing reverse transcription, the obtained cDNA was subjected to RNA concentration detection using NanoDrop (Thermo Fisher Scientific, Waltham, MA, USA) to ensure the quality met the requirement of subsequent experiments. Amplification was performed using TB Green^®^ Premix Ex Taq II (TaKaRa, Japan) on a 7500 Fast Real-Time PCR system (Applied Biosystems, Foster City, CA, USA) and pre-denaturation occurred at 95 °C for 30 s, denaturation at 95 °C for 5 s, annealing at 60 °C for 30 s and conducting over 40 cycles. Melting curves were obtained at 95 °C for 15 s, 60 °C for 1 min and 95 °C for 15 s. The mRNA and miRNA were used as internal reference genes with *GAPDH* and *U6*, respectively. The relative expression levels of the genes were calculated by the 2^−ΔΔCt^ method. The primers (Tsingke Biotechnology, Beijing, China) designed by the laboratory are listed in Table 1.

### 2.7. Western Blot

THP-1 cells were inoculated into six-well plates, induced to differentiate with PMA, and divided into the infected group (MOI = 1), negative control and positive control (LPS and Nigericin treatment) as described previously. Cellular proteins were extracted at the indicated times using RIPA lysate containing 1% PMSF (Solarbio, Beijing, China), the protein concentration was determined using the BCA Protein Assay Kit (Beyotime, China), and then it was mixed with protein loading buffer (Solarbio, China), denatured in a metal bath at 95 °C for 10 min and stored at −80 °C. The samples were added to an SDS-PAGE gel for electrophoresis, then the protein was transferred on the gel to the PVDF membrane (Millipore, Burlington, MA, USA) with membrane transfer buffer (EpiZyme, Shanghai, China) under an ice bath condition. The PVDF membrane was treated with blocking buffer (EpiZyme, China) for 15 min. The membrane was rinsed with TBST three times, each time for 10 min. After rinsing, the PVDF membrane was incubated with the corresponding primary antibody diluted to 1:1000 at 4 °C overnight. The primary antibody included anti-Pro IL-1β (Cell Signaling Technology, Danvers, MA, USA), anti-cleaved IL-1β (Cell Signaling Technology, USA), anti-NLRP3 (ZEN-BIOSCIENCE, Chengdu, China), anti-GSDMD (ZEN-BIOSCIENCE, China), anti-GSDMD-NT (Abcam, UK), anti-caspase-1 (ZEN-BIOSCIENCE, China) and anti-β-actin (ImmunoWay, Newark, DL, USA). On the next day, the PVDF membrane was rinsed three times with TBST for 10 min each time. The proteins were incubated with 1:10000 diluted horseradish peroxidase-labeled secondary antibody Goat Anti-Mouse IgG/Goat Anti-Rabbit IgG (Abcam, UK) for 1 h at room temperature. After incubation, the PVDF membrane was rinsed three times with TBST for 10 min each time. Protein bands were detected using an enhanced chemiluminescence kit (Biosharp, Hefei, China) and a chemiluminescence analyzer (Bio-Rad, Hercules, CA, USA).

### 2.8. Small RNA Sequencing and Analysis

THP-1 cells were inoculated into six-well plates, induced to differentiate with PMA, and divided into the infected group (MOI = 1) and negative control as described previously. Cellular RNA was extracted at 24 h, 48 h and 72 h of infection using the RNeasy Mini Kit. The samples were tested for compliance with library requirements, and then a library was constructed using the NEB Next^®^ Multiplex Small RNA Library Prep Set for Illumina^®^ (NEB E7300L). Library quality was assessed on an Agilent 5400 system (Agilent, Santa Clara, CA, USA) and quantified by qPCR (1.5 nM). After purification and size selection, libraries with insert lengths between 18 and 40 bp were ready for sequencing on the Illumina SE50 platform.

Raw data were filtered using customized perl and python scripts to obtain clean reads without low-quality reads and sequencing junctions. Clean reads from each sample were filtered, sRNAs of 18–40 bp were selected and localized to the reference sequence by bowtie [20], and then the distribution of sRNAs in the reference sequence situation were analyzed. The above reads were mapped to the reference sequences, with miRBase20.0 serving as the reference, and modified software mirdeep2 (mirdeep2_0_0_5) [21] and srna-tools-cli (http://srna-tools.cmp.uea.ac.uk/, accessed on 11 May 2023) were used to obtain the potential miRNA and draw secondary structures. At the same time, new miRNAs were predicted by using the signature hairpin structure of the miRNA precursors. Subsequently, the expression levels of the known and new miRNAs in each sample could be counted and normalized by TPM [22], according to the following: normalized expression = mapped reads/total reads × 1,000,000. Two-by-two differential expression analysis was performed using DESeq R package (1.8.3) based on the negative binomial distribution of DESeq2 [23], and *p*-values were adjusted using the Benjamini and Hochberg method, with a corrected *p* < 0.05 set as the screening condition for significant differential expression by default. The target genes of miRNAs were predicted using two software programs, miRanda (miRanda-3.3a) and RNAhybrid (RNAhybrid v2.0), and the intersection was taken as the final targeting result. R language was used to perform Gene Ontology (GO) and KEGG enrichment analyses on the candidate target genes of differentially expressed miRNAs through the “clusterProfiler” package (3.8.1).

### 2.9. Statistical Analysis

All experiments were performed at least 3 times. Data were analyzed using GraphPad Prism 9.0 software and are expressed as the mean ± standard error of mean. One-way ANOVA was used for multi-group comparison and *t*-test for two-group comparison. A *p* < 0.05 was considered statistically significant.

## 3. Results

### 3.1. DENV-1-Infected Macrophages Cause Cell Death Along with Increased LDH Release

**Figure 1:**
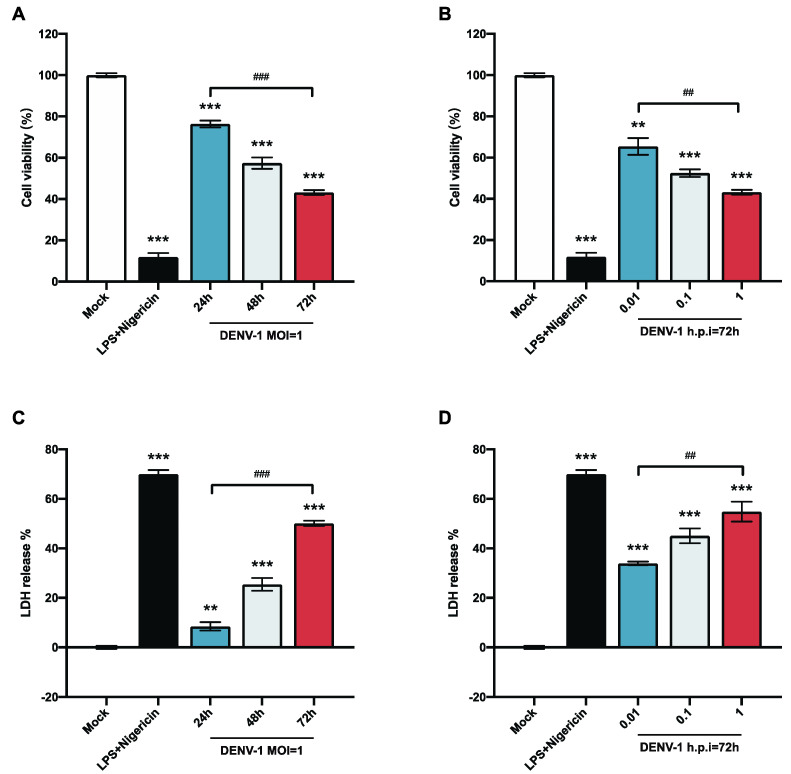
Results of macrophage cell viability and LDH release. (**A**,**B**) Macrophage cell viability was detected using CCK-8; (**C**,**D**) LDH release results were detected by collecting cell supernatants in different culture states. ** *p* < 0.01, *** *p* < 0.001 compared with the negative control group; ^##^ *p* < 0.01, ^###^ *p* < 0.001 compared between the two groups.

To explore the biological alterations of macrophages after DENV-1 infection, the survival rate of macrophages was detected at different viral loads and infection times. The results showed that the survival rate of macrophages infected with DENV-1 was significantly reduced. The macrophage survival rate of the DENV-1-infected group with MOI = 1 and the positive control group (LPS and Nigericin treatment) were significantly lower than those of the negative control group. The proliferation of living macrophages decreased by approximately 20% after 24 h of infection and worsened with the duration of infection (Figure 1A). In the later stage of infection (h.p.i = 72 h), macrophages were significantly damaged even with a low-dose (MOI = 0.01) infection, and the higher the dose of DENV-1 infection, the lower the survival rate of macrophages (Figure 1B).

LDH is an intracellular plasma enzyme that is released extracellularly when the cell membrane is damaged. To further illustrate the alteration of DENV-1 infection on macrophage membranes, the LDH release rate was measured by collecting the cell supernatant. The results showed that DENV-1 infection of macrophages resulted in a significant increase in LDH release compared with the negative control group, and the LDH release rate was positively correlated with the infection dose and infection time (Figure 1C,D), suggesting that DENV-1 infection leads to the rupture of macrophage membranes and the release of contents. The classical pyroptosis pathway involves perforating the cell membrane through the N-terminal fragment of gasdermin D (GSDMD), so we concluded that DENV-1 infection of macrophages may induce pyroptosis [24,25].

### 3.2. DENV-1-Infected Macrophages Release Large Amounts of IL-1β and Promote IL-1β mRNA and Its Protein Expression

**Figure 2:**
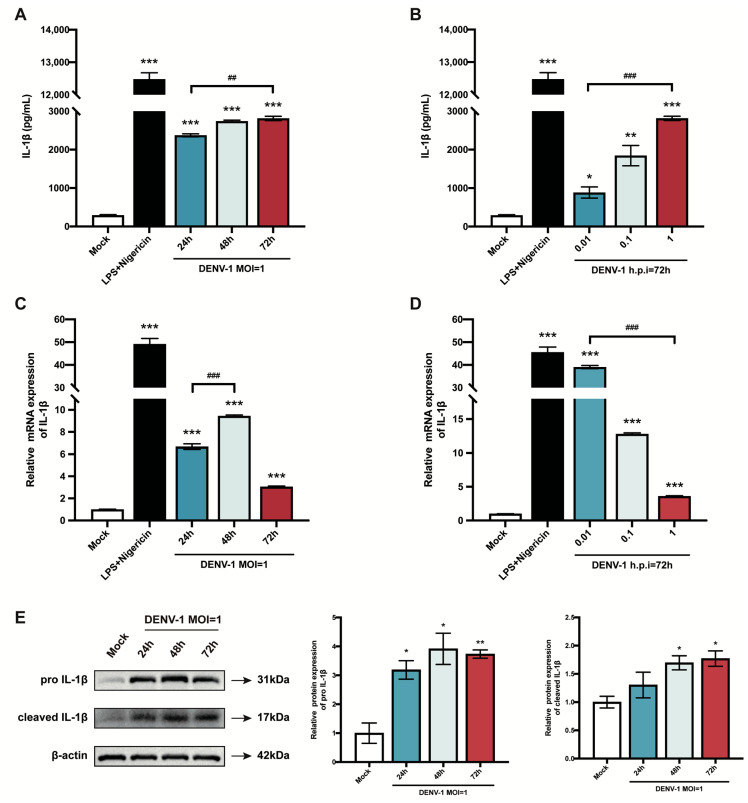
Results of macrophage IL-1β release and mRNA and protein expression level. (**A**,**B**) Supernatants were collected under different culture conditions, and IL-1β content was detected using an ELISA kit; (**C**,**D**) Relative quantification of *IL-1β* mRNA expression level by RT-qPCR. (**E**) IL-1β protein expression levels were detected using Western blot, and β-actin was used as an internal reference control. * *p* < 0.05, ** *p* < 0.01, *** *p* < 0.001 compared with the negative control group; ^##^ *p* < 0.01, ^###^ *p* < 0.001 compared between the two groups.

Pyroptosis is a mode of inflammatory programmed cell death, which results in cell death while releasing the pro-inflammatory cytokine IL-1β extracellularly, exacerbating the inflammatory response [26]. Therefore, this study explored the effect of DENV-1 infection on IL-1β. First, the content of IL-1β secreted in the cell supernatant was measured, and the results showed that the release of IL-1β increased significantly after DENV-1 infection compared with the negative control, with the same trend as the positive control, and increased with the increase in infection time and infection dose (Figure 2A,B). Subsequently, the *IL-1β* mRNA expression level was also examined, which peaked at h.p.i = 48 h in high-dose DENV-1-infected macrophages (Figure 2C). Interestingly, in the later stage of infection, the *IL-1β* mRNA expression level was negatively correlated with the infected dose, which may have been due to the longer duration of action of the virus at lower doses (Figure 2D). In addition, the Western blot results showed that DENV-1 infection resulted in a significant increase in the expression levels of both IL-1β precursors and lysosomes (Figure 2E), which is a characteristic of pyroptosis, further suggesting that DENV-1 infection induced macrophage pyroptosis.

### 3.3. DENV-1-Infected Macrophages Activate NLRP3 Inflammasome, Induce Caspase-1 and GSDMD Activation, and Promote Pyroptosis

**Figure 3:**
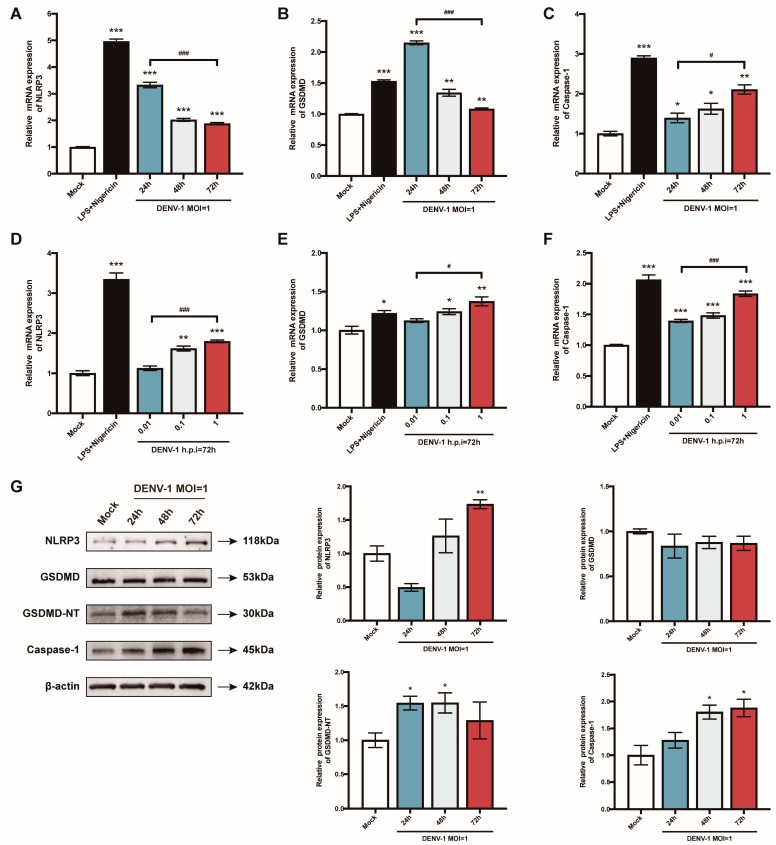
Results of the detection of macrophage pyroptosis-related gene mRNA and its protein expression level. (**A**–**F**) Detection results of the expression levels of pyroptosis-related gene mRNA under different culture conditions; (**G**) The expression levels of pyroptosis-related proteins in macrophages infected with DENV-1 (MOI = 1) for 24 h, 48 h and 72 h, where β-actin was used as an internal reference control. * *p* < 0.05, ** *p* < 0.01, *** *p* < 0.001 compared with the negative control group; ^#^ *p* < 0.05, ^###^ *p* < 0.001 compared between the two groups.

The pyroptosis-related genes were further examined. When macrophages were infected with a high dose of DENV-1 (MOI = 1), *NLRP3* and *GSDMD* mRNA reached a peak value in the early stage of infection (h.p.i = 24 h), while the *Caspase-1* mRNA level gradually increased with the infection time (Figure 3A–C). Meanwhile, the expression of all the pyroptosis-related genes showed a dose-dependent pattern (Figure 3D–F). Protein levels were also measured, and the expression of the NLRP3 inflammasome, GSDMD-NT, and caspase-1 were all enhanced after DENV-1 infection, which further verified that DENV-1 induced macrophage pyroptosis (Figure 3G).

### 3.4. DENV-1 Infection of Macrophages Leads to Changes in miRNA Expression Profiles

**Figure 4:**
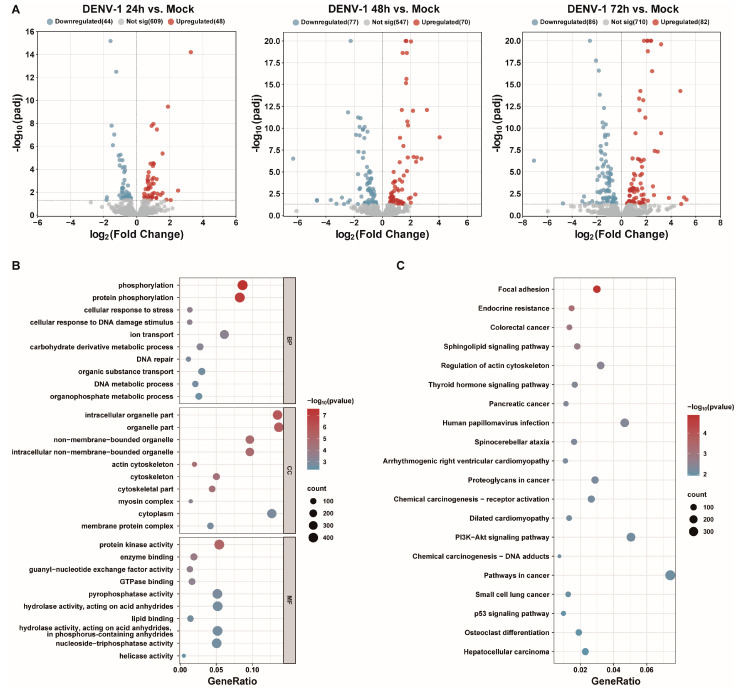
Transcriptome sequencing results and analysis after DENV-1 infection in macrophages. (**A**) Differentially expressed miRNAs in the infected group and the negative control group at different infection times, where blue indicates downregulation and red indicates upregulation; (**B**) GO enrichment analysis of differentially expressed miRNA target genes at h.p.i = 72 h, showing the top 10 entries of difference in each classification; (**C**) KEGG enrichment analysis of differentially expressed miRNA target genes at h.p.i = 72 h, showing the entries in the top 20 differences.

Small RNA transcriptome sequencing was used to obtain the differentially expressed miRNAs at different times after the DENV-1 infection of macrophages. With the increase in DENV-1 infection time, 92, 147 and 168 differentially expressed miRNAs were found at 24 h, 48 h and 72 h, respectively (Figure 4A). Subsequently, the differentially expressed miRNAs screened at an infection time of 72 h were predicted for target genes and subjected to functional enrichment analysis. GO enrichment analysis showed the biological function, pathway and cellular localization of the differentially expressed miRNA-regulated target genes, and the results suggested that the predicted target genes were more related to protein phosphorylation, cellular response to DNA damage stimuli, cytoskeleton, protein kinase activity, etc. (Figure 4B). KEGG enrichment analysis showed that the candidate target genes were mainly involved in actin cytoskeleton regulation, human papillomavirus infection, P13K-AKT signaling pathway, etc. (Figure 4C).

**Figure 5:**
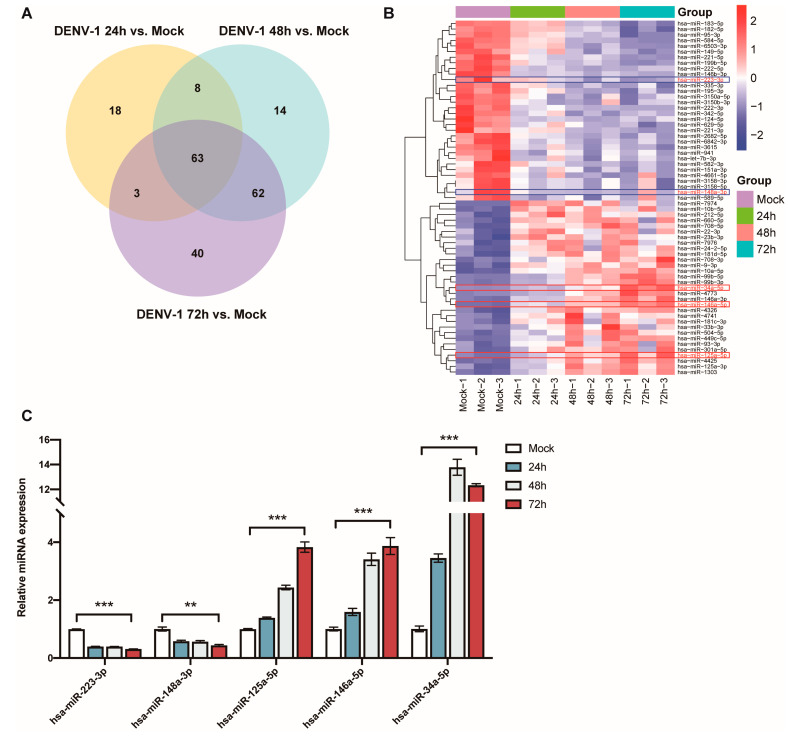
Analysis and validation of differentially expressed miRNAs after DENV-1 infection of macrophages. (**A**) Venn diagram of miRNAs differentially expressed at 24 h, 48 h and 72 h of infection; (**B**) Heatmap of miRNA clustering for miRNAs that were differentially expressed at all infection periods, with the red colored-frame indicating upregulation and blue colored-frame indicating downregulation; (**C**) Detection of the expression levels of *miR-223-3p*, *miR-148a-3p*, *miR-125a-5p*, *miR-146a-5p* and *miR-34a-5p* by RT-qPCR in macrophages at all infection periods. ** *p* < 0.01, *** *p* < 0.001 compared with the negative control group.

The Venn diagram of the differentially expressed miRNAs at the three different periods shows that a total of 63 miRNAs showed significant differences in their expression levels at 24 h, 48 h and 72 h (Figure 5A). This included the downregulation of *miR-223-3p* and *miR-148a-3p*, as well as upregulation of *miR-125a-5p*, *miR-146a-5p* and *miR-34a-5p*, which have been found to be associated with inflammatory responses (Figure 5B).

The expression levels of these miRNAs, which are thought to be associated with inflammation, were detected using RT-qPCR in the DENV-1-infected group (MOI = 1) and in the negative control group, and they were compared and validated with the sequencing results. The results indicated that *miR-223-3p* and *miR-148a-3p* were significantly downregulated in the DENV-1 group, with their expression levels negatively correlated with the infection time. Conversely, *miR-125a-5p*, *miR-146a-5p* and *miR-34a-5p* were significantly upregulated in the DENV-1 group, among which *miR-125a-5p* and *miR-146a-5p* showed a positive correlation between their expression levels and the infection time. Notably, *miR-34a-5p*, which exhibited the most differential expression, reached its peak 48 h after infection and maintained a high level thereafter (Figure 5C). In summary, the miRNA qPCR expression trends were consistent with the sequencing results and significantly different from those in the negative control group.

## 4. Discussion

In this study, we first demonstrated that the DENV-1 infection of macrophages caused a decrease in cell survival rate and membrane disruption, resulting in an increase in LDH release, along with pro-inflammatory cytokines dividing from pro-IL-1β to active IL-1β and massive secretion. Subsequently, we further found that the DENV-1 infection of macrophages activated the NLRP3 inflammasome and caspase-1, cleaved GSDMD to a GSDMD-NT fragment, and combined with cell membrane liposomes to form pores, resulting in the excretion of LDH, IL-1β and other cell contents, causing inflammation and inducing pyroptosis.

Pyroptosis is a pro-inflammatory mode of programmed cell death, which distinguishes itself from apoptosis and autophagy by triggering a series of inflammatory responses [27]. NLRP3, the most extensively studied inflammasome within the NLR family, is associated with a wide range of diseases and functions as a component of its host’s innate immune system. NLRP3 can recognize pathogen-associated molecular patterns (PAMPs) and damage-associated molecular patterns (DAMPs), and it can promote the assembly of an inflammasome complex with the adaptor protein ASC and the effector protease caspase-1, which ultimately lead to pyroptosis [28,29]. Pyroptosis can eliminate viral replication sites within infected cells and mediate innate immune responses in host defense [30]. However, excessive pyroptosis-induced inflammatory responses can result in tissue damage and exacerbate disease progression. For example, Zika virus (ZIKV) infection in mice induces neuronal cell pyroptosis and pathological alterations, leading to brain atrophy, which may be directly associated with microcephaly caused by ZIKV [31]. Furthermore, H7N9 influenza virus-induced alveolar epithelial cell pyroptosis triggers a cytokine storm, thereby increasing the mortality rate of infected mice [32].

Patients with severe DENV infection may present with clinical manifestations such as hemorrhage or even shock, mainly due to the increased permeability of vascular endothelial cells, leading to a large loss of intravascular plasma and albumin. Currently, cytokine storm is considered to be one of the main causes of vascular leakage [33]. Additionally, studies have shown that serum levels of IL-1β in DENV-infected patients are significantly higher than those in uninfected individuals [34]. Further research has revealed that compared to patients with DF, those with DHF produce higher levels of IL-1β [35], suggesting that excessive secretion of IL-1β may influence the clinical progression of the disease. Therefore, in DHF and DSS, we speculate that the large number of pro-inflammatory cytokines released due to pyroptosis may be one of the causes of the increased permeability of vascular endothelial cells and vascular leakage, but the specific mechanism still needs to be further explored.

MiRNAs have the ability to regulate multiple pathways, including inflammatory and innate immune responses [36]. In this study, we found that DENV-1 infection of macrophages caused multiple miRNAs to be differentially expressed, and the changes in miRNAs were further deepened with the extension of infection time. Enrichment analysis revealed that differentially expressed miRNAs predicted target genes highly enriched in protein phosphorylation, stress response and regulation of the cytoskeleton, suggesting that these target genes may be involved in protein activation and a series of antiviral immune responses, which are associated with vascular endothelial cell damage, increased permeability and leakage. In addition, 63 miRNAs with significant differences in the whole process of infection were identified by drawing Venn diagrams of differentially expressed miRNAs at different stages of infection, including miR-223-3p, miR-148a-3p, miR-125a-5p, miR-146a-5p and miR-34a-5p, which have been suggested to be associated with inflammatory responses.

MiR-223-3p is one of the most widely studied inflammatory regulators, with NLRP3 as its direct target, and it is able to negatively regulate inflammasome activation and pyroptosis in inflammatory responses, such as endothelial cell inflammation in syphilis spirochete infections, acute lung injuries and myocarditis [37,38,39]. MiR-148a-3p is also involved in the regulation of inflammation in atherosclerosis and brain injury [40,41]. MiR-125a-5p and miR-146a-5p, which are also reported to be involved in innate immune response and inflammation, have elevated expression in the peripheral blood of patients with rheumatoid arthritis, and they have been considered as biomarkers for detecting rheumatoid arthritis [42,43,44]. In addition, miR-125a-5p was found to promote vascular endothelial cell pyroptosis by inhibiting the DNA demethylase TET2, suggesting that miR-125a-5p is involved in the regulation of vascular function and inflammation [45]. In this study, DENV-1 infection of macrophages caused the most significant upregulation of miR-34a-5p. Previous studies have shown that adipocytes secreting excess miR-34a inhibited macrophage M2 polarization and exacerbated systemic inflammation [46]. Recently, miR-34a-5p was found to induce oxidative stress, inflammation and pyroptosis by negatively regulating the Sirtuin family of proteins (e.g., Sirtuin1, Sirtuin3), whereas inhibition of miR-34a-5p could play a protective role [47,48]. Among these miRNAs, only miR-223-3p and miR-146a have been studied to be involved in regulating DENV infection. miR-223-3p directly targets the microtubule-destabilizing protein stathmin 1 (STMN1) of DENV-2, thereby inhibiting its replication levels [49]. miR-146a promotes the replication of DENV-2 in THP-1 cells by targeting TRAF6 and inhibiting interferon induction. In A549 cells, miR-146a inhibits DENV-2-induced autophagy by targeting TRAF6 [50,51]. However, the relationships between miR-148a-3p, miR-125a-5p and miR-34a-5p with DENV infection have not been confirmed and require further investigation.

Enoxacin is a broad-spectrum antibacterial fluoroquinolone, and it has been proved to be an effective miRNA modulator. Studies have found that enoxacin inhibits the expression of miR-34a-5p in adipocytes, thereby enhancing FGF21 signaling to promote oxidative metabolism and combat obesity [52]. Additionally, based on in silico analysis, enoxacin has been identified as a potential inhibitor of SARS-CoV-2 through the RNAi pathway [53]. Interestingly, enoxacin may also influence the pathogenic mechanisms of DENV by regulating miRNAs.

There were some limitations to this study. Firstly, only DENV-1, which has caused significant human harm and economic losses in Zhejiang Province, was investigated in this study, while the other three serotypes were not studied in depth. Meanwhile, the detection methods could be diversified, such as using electron microscopy to observe cells and detecting other genes and proteins like IL-18 and ASC, which may provide more comprehensive validation of the pyroptotic response. Secondly, the role of miRNAs in regulating DENV-1-induced macrophage pyroptosis has not been verified. Lastly, this study was still confined to cellular experiments in vitro, and the specific molecular mechanisms by which miRNAs affect pyroptosis remain to be further explored.

In summary, our study revealed that DENV-1 induced macrophage pyroptosis and concurrently altered the expression profiles of specific miRNAs, namely miR-223-3p, miR-148a-3p, miR-125a-5p, miR-146a-5p and miR-34a-5p, which are known as small molecules regulating inflammation and pyroptosis. Based on these findings, we hypothesize that these miRNAs may serve as regulators in DENV-1-induced macrophage pyroptosis. Furthermore, verifying the effects of these miRNAs on DENV-1-induced pyroptosis and elucidating its specific mechanism will form the direction of our future research. In conclusion, our study provides a new perspective for exploring the pathogenic mechanism of DENV, suggesting that miRNAs may be a promising therapeutic target for the treatment of DENV-infected patients.

## Figures and Tables

**Table 1:** Primer sequences used in this study.

Genes	Forward Primer (5′-3′)	Reverse Primer (5′-3′)
*IL-1β*	CCAAAGAAGAAGATGGAAAAGC	GGTGCTGATGTACCAGTTGGG
*NLRP3*	GATCTTCGCTGCGATCAACAG	CGTGCATTATCTGAACCCCAC
*GSDMD*	AACTCGCTATCCCTGTTGTCTAC	CCACACTCGTCCAGCAAGAC
*Caspase-1*	TCCAATAATGGACAAGTCAAGCC	GCTGTACCCCAGATTTTGTAGCA
*GAPDH*	AGCCTTCTCCATGGTGGTGAAGAC	CGGAGTCAACGGATTTGGTCG
*miR-223-3p*	GCGCGTGTCAGTTTGTCAAAT	AGTGCAGGGTCCGAGGTATT
*miR-148a-3p*	GCGCGTCAGTGCACTACAGAA	AGTGCAGGGTCCGAGGTATT
*miR-125a-5p*	GCGTCCCTGAGACCCTTTAAC	AGTGCAGGGTCCGAGGTATT
*miR-146a-5p*	CGGCTGAGAACTGAATTCCA	ACTGCAGGGTCCGAGGTATT
*miR-34a-5p*	CGCGTGGCAGTGTCTTAGCT	AGTGCAGGGTCCGAGGTATT
*U6*	CTCGCTTCGGCAGCACA	AACGCTTCACGAATTTGCGT

## Data Availability

All data are provided within the manuscript.
